# Northstar enables automatic classification of known and novel cell types from tumor samples

**DOI:** 10.1038/s41598-020-71805-1

**Published:** 2020-09-17

**Authors:** Fabio Zanini, Bojk A. Berghuis, Robert C. Jones, Benedetta Nicolis di Robilant, Rachel Yuan Nong, Jeffrey A. Norton, Michael F. Clarke, Stephen R. Quake

**Affiliations:** 1 Department of Bioengineering, Stanford University, Stanford, CA USA; 2 Prince of Wales Clinical School and Adult Cancer Program, UNSW, Sydney, Australia; 3 Department of Oncology, Stanford School of Medicine, Stanford, CA USA; 4 Department of Immunology, Genetics and Pathology and SciLifeLab, Uppsala University, Uppsala, Sweden; 5 Department of Surgery, Stanford Pancreas Cancer Research Group, General Surgery, Stanford University School of Medicine, Stanford, CA USA; 6 Department of Applied Physics, Stanford University, Stanford, CA USA; 7 Chan Zuckerberg Biohub, San Francisco, CA USA

**Keywords:** Computational biology and bioinformatics, Classification and taxonomy, Data integration, Functional clustering, Machine learning, Modularity, Cancer screening, CNS cancer, Endocrine cancer, Skin cancer, Tumour heterogeneity

## Abstract

Single cell transcriptomics is revolutionising our understanding of tissue and disease heterogeneity, yet cell type identification remains a partially manual task. Published algorithms for automatic cell annotation are limited to known cell types and fail to capture novel populations, especially cancer cells. We developed northstar, a computational approach to classify thousands of cells based on published data within seconds while simultaneously identifying and highlighting new cell states such as malignancies. We tested northstar on data from glioblastoma, melanoma, and seven different healthy tissues and obtained high accuracy and robustness. We collected eleven pancreatic tumors and identified three shared and five private neoplastic cell populations, offering insight into the origins of neuroendocrine and exocrine tumors. Northstar is a useful tool to assign known and novel cell type and states in the age of cell atlases.

## Introduction

The widespread adoption of single-cell transcriptomics has led to a growing body of “atlas” datasets describing tissue heterogeneity with unprecedented resolution^1^. As atlas data across species^2^, tissues^3–7^, development^8–10^, and aging^4,11^ are being amassed, a number of algorithms have been proposed to compare new data against reference data sets. The basic concept behind these efforts is to emulate the Basic Local Alignment Search Tool (BLAST) for nucleic acid and protein sequence matching^12^, except in gene expression space.

Most current approaches including scVI^13^ and scmap^14^ focus on data projection, also known as harmonization, i.e. on embedding the new cells into a neighborhood graph and/or a latent space hosting cells of known type. Although many kinds of analyses are possible after harmonization, many biomedical researchers are most interested in cell type annotation, i.e. the assignment of each new cell into a known cell type or novel cluster. Perhaps surprisingly, no software tool is specifically designed to assign cells to known types while clustering the unassigned cells into novel clusters.

The simplest existing approach (e.g. scmapCell) is to analyse each cell to be annotated independently. For each new cell, its most similar cells in the atlas are found and a consensus rule on their annotations can be used to guess the type of the new cell too. This design is straightforward but unable to find novel clusters, since new cells are never compared to one another.

To give the algorithm a chance to identify novel cell types, a common approach (e.g. scVI) is to first harmonise the new data with the full atlas and then apply unsupervised clustering (e.g. Louvain^15^ and Leiden^16^). Unfortunately, unsupervised clustering depends on a number of subtle parameters (e.g. feature selection, number of dimensions, resolution parameter) and is not aware of the annotations at all; these factors often cause inconsistent clustering of the atlas itself, leading to poor predictions. For instance, if atlas cells from two distinct types get clustered together—which can happen routinely in practice—classifying the new cells becomes a very confusing task.

An alternative approach, shared by scClassify^17^ and scmapCluster (part of scmap) is to either assign new cells to a known type or reject them into an “unknown” bin. This route ensures a consistent annotation, however it fails to identify subclusters within the “unknown” category. Ad-hoc unsupervised clustering of the “unknown” bin is possible (e.g. scClassify), yet the choice of parameters is again arbitrary and can lead to inconsistent results. This is an especially severe limitation for tumor samples as the key question is not to confirm the presence of “unknown” neoplastic cells—that is already known by histology—but to identify subpopulations that might be more or less malignant.

These issues share a common origin: the clustering algorithm is unaware of the cell atlas annotations. To address this issue, we have developed northstar, an algorithm and software package that classifies single-cell transcriptomes guided by training data but is also able to discover new cell types or cell states. The inspiration for northstar comes from harmonization^13,14^ and batch correction techniques^18,19^, however northstar uses an improved clustering step that is aware of the atlas annotations, making it more accurate, faster, and more easily interpretable than other approaches. To benchmark northstar we analyzed published datasets on glioblastoma^20^, melanoma^21^, and autism^22^ and found a superior ability to classify known and cluster novel cell types in actual tumor samples. We then evaluated northstar on datasets with more than 10,000 cells and observed higher accuracy than scVI and scmap. We subsequently applied northstar to newly collected 1,622 cells from 11 pancreatic cancer patients and rapidly classified them into healthy cell types and neoplastic states and ultimately gained useful biological insight into the composition and origin of the tumors.

## Results

### Northstar identifies cell types guided by a cell atlas

Northstar is a computational approach to identify cell types in a new single cell dataset leveraging one or multiple cell atlases. The unique feature of northstar is that every new cell can be either assigned to either a known atlas cell type or a novel cluster. An implementation in C++/Python is available at https://github.com/northstaratlas/northstar and preprocessed atlases for immediate use are available at https://northstaratlas.github.io/atlas_landmarks.

As input, the gene expression table for the new dataset and the gene expression table and cell type annotations for the cell atlas are specified by the user. Northstar can use either an average expression vector for each cell type or a small subsample of each cell type. Atlas averages and subsamples are approximate, compressed representations of the atlas and can be used by northstar as *reference landmarks* to annotate the new cells. In this sense, northstar serves the same purpose in single-cell datasets as the North Star always had for maritime navigation: providing fixed points that guide rather than limit the exploration of new landscapes. To simplify adoption, we provide precomputed landmarks (averages and subsamples) of several atlases (see above link). If a precomputed atlas is chosen, the user only needs to specify its name: counts and annotations are downloaded automatically.

**Figure 1:**
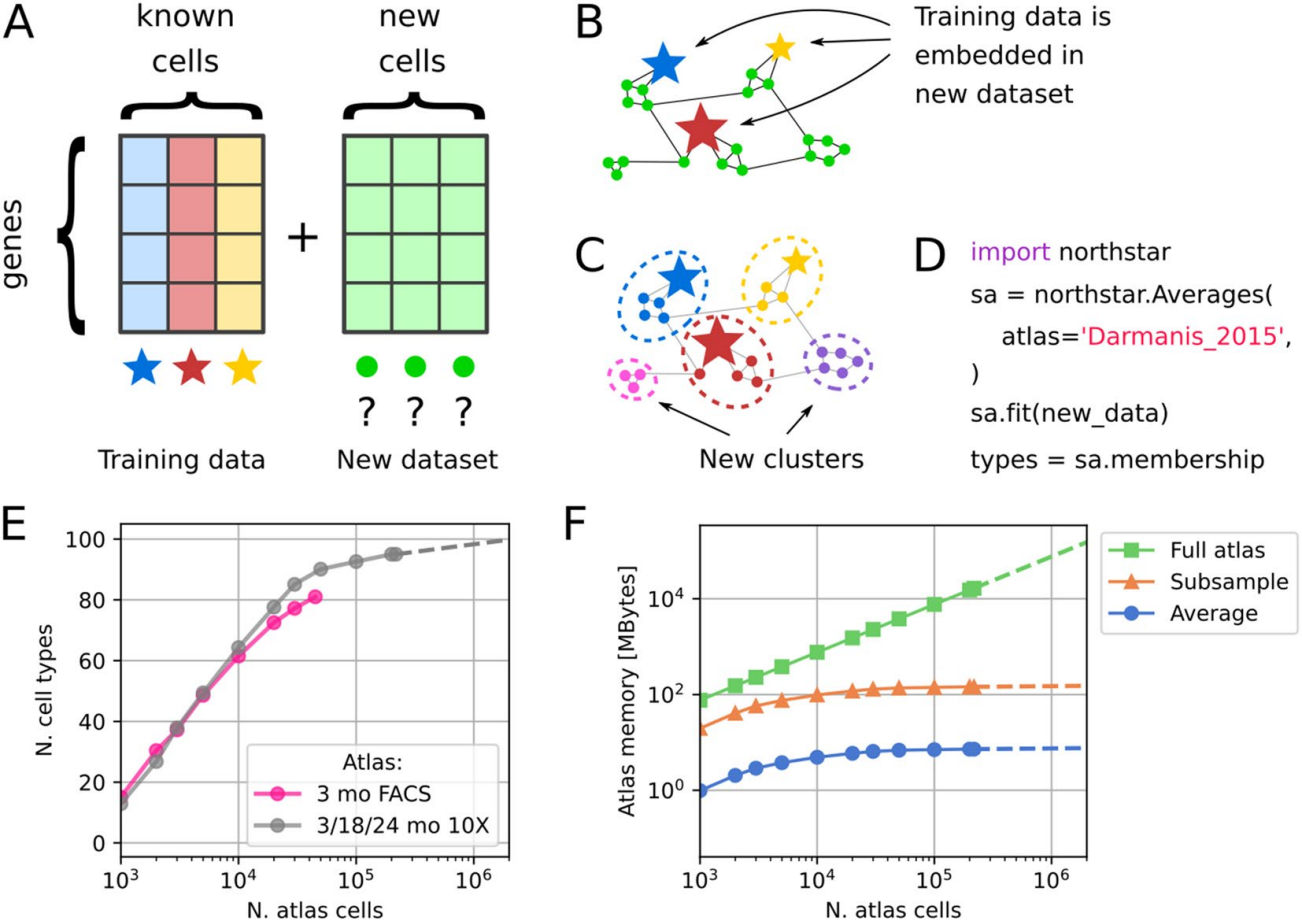
Northstar concept and scalability. (**A**) Northstar’s input: the gene expression table of the tumor dataset and the cell atlas. Annotated cell type averages are depicted by colored stars, unannotated new cells by green circles. (**B**) Similarity graph between atlas and new dataset. (**C**) Clustering the graph assigns cells to known cell types (stars) or new clusters (pink and purple, bottom left and right). Cell types themselves do not split or merge. (**D**) Typical code used to run northstar. (**E**) Number of cell types with at least 20 cells in Tabula Muris (FACS data, pink) and Tabula Muris Senis (10 ×/droplet data, grey), subsampled to different sizes^2, 11^. (**F**) Memory needed to store the Tabula Muris Senis atlas, subsampled to different sizes as in E, as a full atlas and using the two approaches within northstar. Subsample assumes 20 cells per cell type. Memory for the new dataset to be annotated should be added to this footprint independently of the classification algorithm.

The algorithm consists of the following steps. First, atlas landmarks (averages or subsamples) are merged with the new single-cell dataset into a single data table (Fig. 1A). Then, informative genes are selected: upregulated markers of each atlas cell type are included as well as genes showing a high variation within the new dataset. A similarity graph of the merged dataset is constructed, in which each edge connects either two cells with similar expression from the new dataset or a new cell with an atlas cell type (Fig. 1B). Finally, nodes in the graph are clustered into communities using a variant of the Leiden algorithm that prevents the atlas nodes from merging or splitting^16^. The output of northstar is an assignment of each cell to either an atlas cell type or, if a group of cells show a distinctive gene expression profile, to a novel cluster (Fig. 1C). The clustering step is performed in a separate class called ClusterWithAnnotations which enables combing northstar with data harmonisation techniques via a custom similarity graph^13,18^.

Northstar is designed to be easy to use (Fig. 1D) and scalable. To examine its scalability to large atlases, we downloaded the Tabula Muris plate data^2^ and the droplet Tabula Muris Senis data^11^, subsampled it to different cell numbers, and counted the number of cell types with at least 20 cells. As more cells were sampled, new cell types were discovered, however with diminishing returns. At full sampling (~ 200,000 cells), we estimated that 5 new cell types are discovered per tenfold increase in cell numbers (Fig. 1E). Because of this sublinear behaviour, northstar’s atlas compression design scales to atlases of arbitrary size, unlike a naive approach that combines all atlas cells with the new dataset (Fig. 1F). Although subsampling each cell type (e.g. 20 cells) requires more storage memory than a single average, their scaling behaviour is exactly the same (i.e. logarithmic or better).

### Benchmark against published datasets on healthy brain and glioblastoma

**Figure 2:**
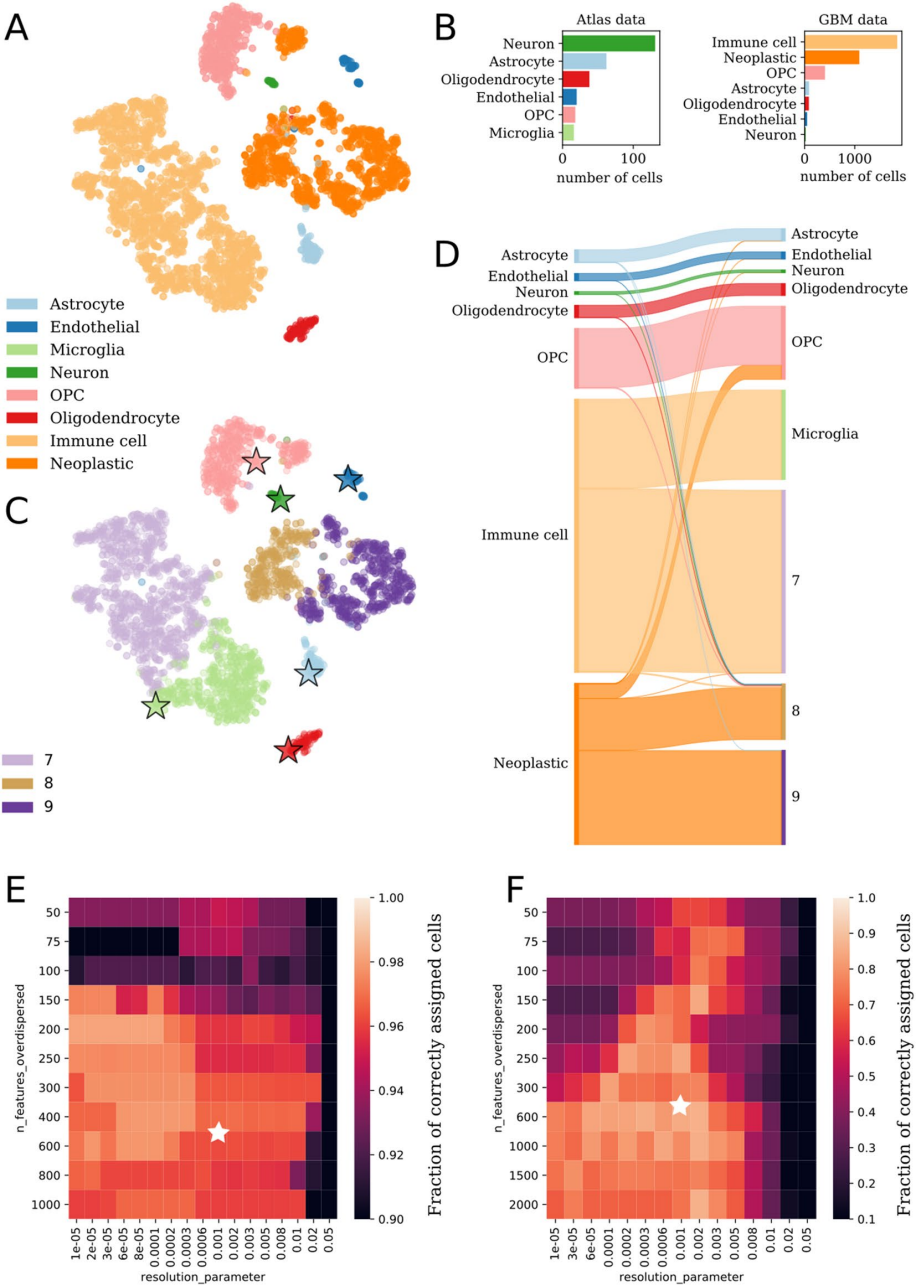
Annotation of glioblastoma and melanoma. (**A**) t-SNE of all cells in Darmanis et al. (2017) colored by original annotation^20^. (**B**) The number and type of cells that constitute the atlas data and the new glioblastoma data. (**C**) The same embedding of glioblastoma cells as in (**A**) but colored by northstar’s annotation and showing atlas cells (stars: atlas averages). (**D**) Sankey diagram with original manual annotations (left) vs. automated annotation by northstar (right). Band widths are proportional to the number of cells in the cluster. (**E**,**F**) Fraction of correctly assigned cells to known cell types (**E**) in the GBM dataset^20^ (mean of 3 northstar runs) and (**F**) in an independent study on melanoma in mice^21^ (mean of 5 runs) upon variation of the number of overdispersed features from the new dataset and the resolution parameter of the Leiden clustering. White stars indicate default parameters.

To validate northstar’s performance, we analyzed a glioblastoma (GBM) dataset^20^ on the basis of a previously annotated cell atlas of the human brain by the same authors^3^. The annotations of the GBM dataset according to the original authors define seven healthy cell types: neurons, astrocytes, oligodendrocytes, oligodendrocyte progenitor cells (OPCs), endothelial cells, microglia, and other immune cells. In addition to these seven, an additional cluster of neoplastic cells is described. Of these cell types, the first five are also present in the brain atlas, while some fetal cells were excluded from the atlas because the GBM patients were adults. A projection of the GBM data via t-Distributed Stochastic Neighbor Embedding (t-SNE) is shown in Fig. 2A with the original annotations (see Supplementary Fig. 1A for the original coordinates: the two maps differ slightly because of feature selection and stochasticity of the t-SNE algorithm). The relative abundances of the various cell types in the atlas and GBM data is shown in Fig. 2B.

We calculated cell type gene expression averages of the brain atlas, deleted the annotations from the GBM data, and fed labeled atlas averages and unlabeled GBM data into northstar. In less than 9 s of runtime on a modern laptop, we obtained a classification of the new cells and observed that our method recapitulated previously reported cell types and also created new classes for myeloid and neoplastic cells as these cell types were absent from the atlas (Fig. 2C, Supplementary Fig. 1).

A detailed analysis of the original annotations versus new annotations generated by our algorithm highlights the strength of the method (Fig. 2D). Almost all cells of known types were correctly assigned to their respective cluster. The main misclassification was a population annotated as neoplastic in the original paper but classified as oligodendrocyte precursor (OPC) by northstar. However, those cells formed a relatively distinct cloud in both Fig. 2C and, even more clearly, in the original t-SNE plot (Supplementary Fig. 1). Moreover, a diverse population of immune cells is either classified as microglia, which are resident immune cells, or into new cluster 7. Neoplastic cells were correctly assigned to new clusters 8 and 9. To identify why both immune and neoplastic clusters were split by northstar we examined their connectedness in the similarity graph. We found that their subcomponents were only weakly connected (Supplementary Fig. 2); the Leiden algorithm—which wasn’t available to Darmanis et al. in 2017—split them to increase internal connectedness^16^. To understand at a broad level the identity of the new clusters, we performed differential expression analysis and found that cluster 7 upregulates CD163 and other immune cell markers, as expected, while the most upregulated genes in the neoplastic cell clusters expressed RNAs previously implicated in malignancies such as TSPAN31 (cluster 8) and TNC (cluster 9).

To test northstar’s accuracy and stability, we repeatedly classified the glioblastoma dataset in 10 independent runs with default parameters and found that in average 97% of cells were correctly classified with a standard deviation between runs smaller than 1%, indicating both high accuracy and stability. Similar classifications were obtained using northstar with a subsample of the atlas, confirming that the classification is robust (Supplementary Fig. 3). To test northstar’s sensitivity to the input parameters, we performed thousands of runs—using an atlas subsample—covering a wide range of the following parameters: (1) number of marker genes per cell types, (2) number of overdispersed features from the new dataset, (3) number of principal components retained, (4) number of graph neighbors, and (5) resolution parameter of the Leiden clustering. We found that only (2) and (5) had any detectable impact on the fraction of correctly assigned cell types, and that our classification was very accurate as long as the number of overdispersed features from the new dataset is at least 150 (to enable sufficient discrimination) and the resolution parameter is less than 0.01 (to avoid subcluster splitting) (Fig. 2E). To exemplify the effect of a relatively high resolution parameter compared to Fig. 2A–D, we also plotted the confusion matrix in a typical northstar run (Supplementary Fig. 4A). Adding the fetal data to the atlas did not have a major effect on the result (Supplementary Fig. 4B).

**Figure 3:**
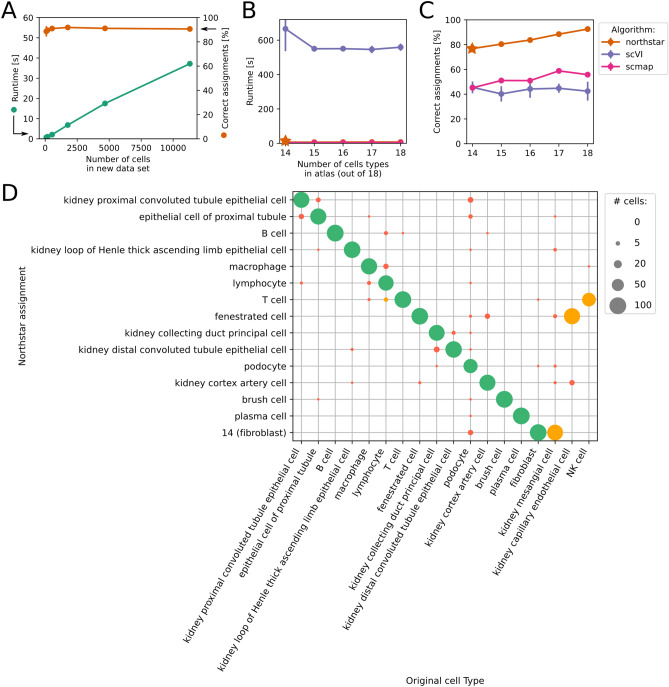
Performance on large datasets and comparison with scVI and scmap on murine droplet kidney data. (**A**) Runtimes and percentage of correctly classified cells with increasing numbers of cells in the new dataset. (**B**) Runtime and (**C**) percentage of correctly assigned cells for northstar, scVI, and scmap with an incomplete atlas containing only a fraction of the 18 cell types. Northstar is much faster since it does not need to train a deep neural network. Northstar is also more accurate because of its atlas-aware clustering step. All algorithms used the same input data (1,766 cells as test, spread evenly across cell types). Northstar’s accuracy surpasses 90% as the atlas becomes more complete. (**D**) Typical confusion matrix of a northstar run with an incomplete atlas [star in (**B**) and (**C**)]. Most cells are classified correctly (green dots) or assigned to similar cell types (yellow dots), while a small number of cells are misclassified into a distinct cell type (red dots). Subsamples of the kidney droplet data from Tabula Muris Senis^11^ with 20 cells per type were used as atlas.

**Figure 4:**
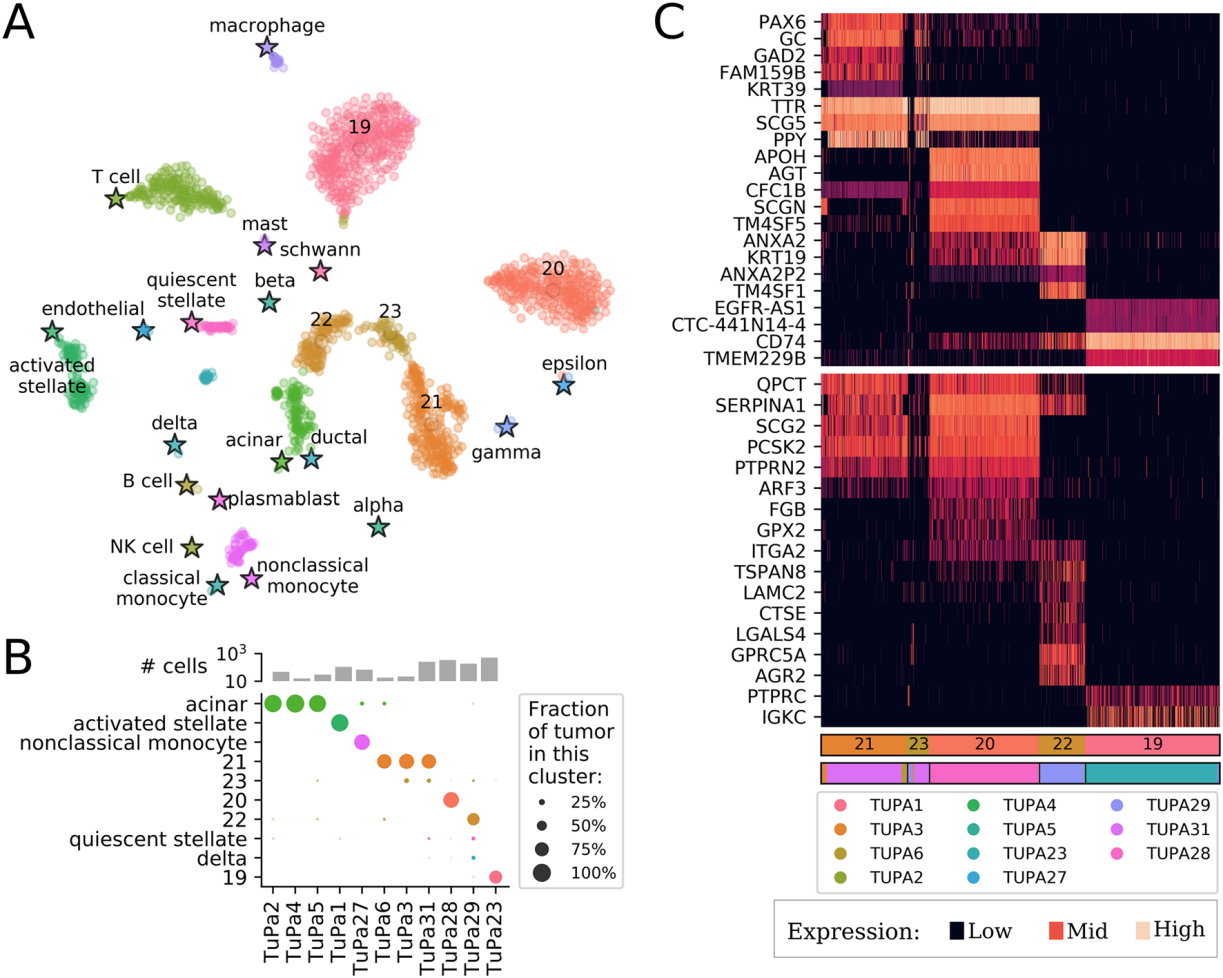
Annotation of new pancreatic tumor samples. (**A**) t-SNE of 11 pancreatic tumors together with averages from two atlases by Baron et al. (2016) and Zanini et al. (2018)^24,25^. Stars: atlas averages. (**B**) Number of cells from each tumor and fractions of cells belonging to each cell type. (**C**) Top differentially expressed genes for each novel cluster (top) and some known markers (bottom) for pancreatic cancers^38^. Black indicates no expression, red to white increasing expression levels. PTPRC (CD45) and IGKC are added to highlight the probable B cell phenotype of cluster 19. First bar: novel cluster [colored as in (**A**) and (**B**)]. Second bar: patient of origin, with legend below.

To test northstar’s classification across wide technical biases between atlas and new data, we computed a subsample of another human brain dataset on autism (ASD) by Velmeshev et al.^22^, which had been prepared via droplet-based libraries. We found that northstar is just as accurate with the Velmeshev atlas, despite a different laboratory and library technology (Supplementary Fig. 4C). The use of control individuals from the Velmeshev dataset as an atlas for their ASD patients resulted in a good classification as well (Supplementary Fig. 4D,E). Notice that northstar uses a separate class called ClusterWithAnnotations to perform the clustering step: this modular design enables advanced users to combine more sophisticated data harmonization methods to build a cell similarity graph with northstar’s atlas-aware clustering algorithm.

To test northstar’s performance on a larger cancer dataset, we analyzed 4,626 cells from a melanoma murine model focused on infiltrating immune cells^21^. As with the GBM data, we found high accuracy with the default parameters (~ 90% correctly assigned immune cells using Tabula Muris marrow annotations only). For this dataset, selecting 600 or more features from the new dataset generally increased accuracy (Fig. 2F). In terms of speed, northstar classified the > 4,000 cell melanoma dataset in approximately 9 s on a modern laptop using a single low-voltage CPU core (Supplementary Fig. 5). These findings highlight northstar’s core concept that even just a few example transcriptomes for each cell type are sufficient to guide the classification task robustly and efficiently.

### Performance of northstar on Tabula Muris Senis and comparison with other tools

We then proceeded to assess northstar’s performance on droplet data from Tabula Muris Senis, which includes more than 200,000 cells across tissues, ages, and both genders. We first focused on kidney data and created a subsample of the atlas with only 20 cells per cell type. We then used it as a guide to annotate larger subsamples of the same dataset, up to around 12,000 cells (Fig. 3A). To ensure that the performance evaluation was not skewed by a few common cell types, “new data” sets were constructed by sampling the same number of cells from each type. We found that northstar’s runtime increases approximately linearly with the size of the dataset to annotate (green line, left axis) while the percentage of correctly assigned cells is constant around 90%. A similar result was found across several other tissues, although some tissues such as bone marrow had lower accuracies, perhaps due to a more subtle cell type structure (Supplementary Fig. 6). Notice that the runtime calculation includes not only the clustering but also class initialization, merging the atlas and new data, joint normalization, defining markers for known cell types, identifying overdispersed features in the new data, feature selection, dimensionality reduction, and graph construction.

Northstar is designed to perform in the face of an incomplete atlas. To recapitulate that scenario, we repeated the previous classification, however this time we removed from the subsampled atlas up to four cell types out of 18 in total (Fig. 3B,C, orange lines). We observed that less complete atlases did not affect northstar’s speed, however accuracy decreased slightly. To compare northstar with related state-of-the-art algorithms, we then performed the same classification task using scVI^13^ and scmap^14^, two popular packages for single cell analyses. Northstar was around 100 times faster than scVI and similarly fast as scmapCell from scmap, while being also much more accurate than either (Fig. 3B,C, purple lines). The difference in runtime with scVI is obvious when running the comparison: scVI uses deep learning and therefore spends minutes training the neural network even with only hundreds of cells. The accuracy discrepancy is also expected because scVI and scmapCell are not per se clustering algorithms but rather data harmonization tools. scVI relies on Leiden for clustering^13,16^ and often splits the atlas clusters into subclusters or mixes them, leading to worse annotations. Although northstar is also based on Leiden, it is further taylored to exploit the information in the incomplete atlas, which guarantees that the atlas clusters will never be split or merged. scmapCell produces a list of neighbors in the atlas and a consensus rule can be used to assign a cell type. While northstar also produces a neighborhood graph, unlike scmapCell neighbors are not limited to atlas cells but can include cells from the new dataset. Moreover, northstar’s atlas-aware clustering step is more robust than a consensus rule, because it exploits information beyond nearest neighbors. These design choices lead to more robust and faithful annotations.

To understand in detail what kind of mistakes are generated by northstar, we focused on the most incomplete atlas with 4 cell types missing (Fig. 3B,C, orange stars) and plotted the confusion matrix for a typical northstar run (Fig. 3D). We noticed that most cells are classified correctly and a new cell type for mesenchymal cells (cluster 14) is correctly created (green dots). Northstar’s inaccuracies were mostly caused by insufficient separation of similar cell types (yellow dots). For instance, northstar assigned natural killer (NK) cells, one of the four cell types removed from the atlas, to T cells, a transcriptionally similar cell type that was still present in the atlas. An analogous mistake led to misclassification of capillary endothelial cells as fenestrated cells and to the merging of fibroblasts and mesangial cells—both mesenchymal cells—into the same novel cluster (14). Northstar misclassified a small number of cells to an unrelated cell type (red dots).

### Classification of healthy and neoplastic cells in pancreatic cancer

To test the ability of northstar to provide biological insight into new datasets, we collected 1,622 single cells from 11 pancreatic tumors (Supplementary Table 1) and aimed to determine their composition in terms of known cell types and, potentially, novel clusters. Briefly, tumors were surgically resected at Stanford hospital from patients with diagnosed pancreatic cancer and dissociated into single-cell suspensions (see “Methods”). Single cells were isolated by fluorescence-activated cell sorting (FACS) into 96- or 384-well microtiter plates and processed as described previously^4,23^.

Northstar was then used to identify the cells in these samples based on the pancreas atlas by Baron et al. (2016)^24^ and the immune atlas by Zanini et al. (2018)^25^. We found that the cells from the cancer patients were classified into both known and novel cell types (Fig. 4A and Supplementary Fig. 7). Among the known cell types were mesenchymal, exocrine and endocrine cell types, and various immune cells. Although all tumors were resected in a similar procedure, the fraction of cells belonging to each known cell type varied considerably among patients (Fig. 4B and Supplementary Table 2). This reflects the challenges of isolating tumor tissue during surgery without capturing surrounding tissue, and making single-cell suspensions from pancreatic exocrine tissue while the tissue is self-digesting.

Samples TuPa2, 4, and 5 were composed mostly of acinar cells—or cells that closely resemble that cell type. In fact, it is clear from the embedding (Fig. 4A) that many of these cells are close to both acinar and ductal types. This was expected for patients TuPa2 and 4 because the tumor was diagnosed as Pancreatic Ductal Adenocarcinoma (PDAC) but not TuPa5, which is annotated as a neuroendocrine tumor. TuPa1 was clinically described as fibromatosis and we found it is essentially composed of activated stellate cells. This is consistent with recent literature^26–28^. Sample TuPa27 was classified as composed mainly of monocytes, which was surprising considering that it was clinically described as a neuroendocrine tumor. Because surgery took longer than usual for this sample, we speculate that the tumor cells might have degraded leaving only the more robust immune cells. All other samples classified in one or more novel clusters. TuPa6, 3, and 31 shared cluster 21 though those patients were diagnosed with three different conditions: ampullary adenocarcinoma, mucinous cystic neoplasm, and neuroendocrine tumor, respectively. The embedding confirms northstar’s prediction and places these cells somewhere between alpha and gamma/PP endocrine types, indicating an endocrine origin. A minority of cells in samples TuPa3 and 31 belonged to another new cluster, 23, which is located nearby on the embedding. The tumor from TuPa28 belonged to its own private cluster 20. Its location is indicative of an endocrine lineage, in agreement with the patient’s diagnosis of neuroendocrine tumor. The donor of TuPa29 was the only one diagnosed with invasive adenosquamous carcinoma and was found to contain a majority of cells in a new, almost private cluster 22 located in the vicinity of acinar cells. A minority of cells from this sample were assigned to quiescent stellate and delta cells. Finally, sample TuPa23 was composed entirely of quasi-private cluster 19, which is surrounded in the embedding by resident immune cells.

To better understand the nature of the novel clusters 19–23, we computed differentially expressed genes (DEGs) between each novel cluster and all other cells by Kolmogorov–Smirnov statistics on the expression. In short, we searched for genes that are expressed by a high fraction of the cells within the focal cluster and by few cells outside of it (Fig. 4C, top heatmap and Supplementary Table 3). The expression of PPY by clusters 21 and 23 is suggestive that these might be neoplastic cells derived from gamma/PP precursors. Clusters 21, 23, and 20 all express TTR and SCG5 but the latter cluster is missing PPY; this favors a distinct endocrine origin for the latter cluster. Cluster 22 expresses KRT19, indicating an epithelial origin which is consistent with their proximity on the t-SNE with acinar and ductal cells. Finally, cluster 19 expresses CD74 which is part of the MHC class II machinery found in immune, antigen presenting cells. To further validate these results, we also looked at the expression of known markers for endocrine and exocrine cancers (bottom heatmap) and found that clusters 20, 21 and 23 show an expression consistent with endocrine cancer cells, while cluster 22 is consistent with exocrine cancer. Cluster 19 expresses PTPRC and IGKC, indicating it is related to B cells. Its location on the embedding supports an immune cell type, although the atlas B cells and plasmablasts are not located in proximity. This discrepancy might be due to biological differences between tumor infiltrating B cells and peripheral blood lymphocytes from healthy subjects.

## Discussion

Annotating a new single-cell transcriptomic dataset traditionally involves unsupervised clustering without an atlas, however this process is laborious and can be inaccurate. Geometric subsampling of the atlas^29^, followed by merging with the new data and unsupervised clustering is a viable route, however known cell types can split into subclusters or merge into superclusters, greatly complicating the interpretation. In our experience such cases happen often because clustering can be performed at different resolutions leading to equally valid classifications (e.g. all immune cells, lymphocytes, T cells). Northstar improves over these approaches by combining cell-type aware subsampling of the atlas at the desired clustering resolution with an atlas-aware clustering algorithm^16^. Known cell types can neither split nor merge simply because they are fully determined by the atlas.

Northstar is efficient because it approximates a cell atlas by compressed representations, i.e. averages or small subsamples. One can easily use an atlas with millions of cells on a laptop with 16 GB of RAM as long as the number of *cell types* remains within a few hundreds. Current atlases only have tens (Figs. 2, 3, 4) of cell types and the current gain is 5 new cell types per tenfold increase in cell numbers (Fig. 1E). Classifying cells is even faster than computing their embeddings (e.g. t-SNE^30^, UMAP^31^) and a systematic benchmark on Tabula Muris Senis indicated a linear scaling of runtime versus new dataset size (Fig. 3A), with absolute runtimes much faster than scVI^13^ because northstar needs not train any neural network (Fig. 3B,C).

In terms of misclassifications, northstar is mostly confused about declaring a novel cell type if a similar one is already present in the atlas, whereas gross misclassification were not observed (Fig. 3D, Supplementary Fig. 4). Northstar is not much affected by batch effects (e.g. droplet versus plate data, Supplementary Fig. 4C). Nonetheless, inspired by the highly modular landscape of UNIX tools, northstar was designed to be compatible with external data harmonisation^13,14,18^ (see^32^ for a systematic comparison) by allowing for a custom similarity graph (Fig. 1B), which is then clustered via northstar’s atlas-aware algorithm (Fig. 1C, class ClusterWithAnnotations).

Unfortunately, many cell atlases are poorly disseminated. Data access is idiosyncratic to each dataset and often requires manual steps (e.g. writing emails to the authors). Cell type annotations are difficult to find and often of mediocre quality, presenting a challenge in particular to algorithms such as scClassify that rely on entire cell type hierarchies^17^. Although northstar is less affected because it does not require a hierarchical ontology, we aspire to change this trend by providing a website with averages and subsamples for several atlases that can be accessed both by humans and programmatically: https://northstaratlas.github.io/atlas_landmarks. This makes it easy to combine atlases and cherry pick different cell types from each to maximize the leverage provided by the annotations. Contributions and requests are welcome.

The analyses of brain and pancreatic tumors presented here highlights the utility of northstar to quickly characterize the cell type composition of tumors. Simple differential expression can be applied immediately afterwards (Fig. 4C) to identify the nature of the new clusters and to shed light on their biological origins. Sampling human tumors is challenging due to cell death preferential to certain cell types (e.g. neurons, pancreatic exocrine cells), hence northstar is an ideal tool to verify whether the cell types of interest are captured effectively. Moreover, the joint analysis of multiple patient samples is informative about how stereotypic neoplastic cell state progression is across individuals. In the 11 pancreatic tumors analyzed, we observed both shared and private clusters and also found corroborating evidence linking fibromatosis to activated stellate cells^26–28^. Cell atlases from large numbers of cancers are being collected in addition to healthy tissues^33^. This will further boost the utility of northstar for rapidly classifying tumors into known or novel neoplastic cell states.

Cell atlases provide an invaluable resource to study heterogeneous disease and in particular cancer. Northstar’s unique ability to identify healthy and neoplastic cells is an important step towards personalized diagnosis and characterization of disease states at the single cell level.

## Methods

All methods were carried out in accordance with relevant guidelines and regulations.

### Brain atlas, glioblastoma dataset, and pancreas atlas

Gene expression count tables and cell type annotations were downloaded from NCBI’s Gene Expression Omnibus website (brain atlas: GSE67835, glioblastoma: GSE84465, pancreas atlas: GSE81547). To combine the brain atlas and glioblastoma dataset, only genes that were present in both datasets were retained. Gene expression counts were normalized by 1 million total counts per cell. For the brain and pancreas atlases, arithmetic averages of the normalized counts were computed for each cell type and stored. Fetal cell types were excluded from the brain atlas since the glioblastoma dataset refers to adult patients, while ambiguous cell types (e.g. “unknown”, “hybrid”) were excluded from both atlases.

Differential expression was computed as follows. For each of the new clusters, expression of all cells within the cluster was averaged and compared to the average of all cell outside the cluster (one-vs-rest). The genes with the 50 highest fold changes were shortlisted and tested for a distribution-level two-sample Kolmogorov Smirnov test at the single cell level with the same grouping. Genes with the largest KS statistic were identified.

### Pancreatic cancer dataset

A novel single-cell transcriptomic dataset from human individuals with pancreatic cancer was collected. Eleven individuals were sampled with a total of 1,622 cells. A table of individual metadata is available as Supplementary Table 1.

Pancreatic tumor tissue was obtained at the Stanford Hospital from individuals undergoing surgery for pancreatic cancer between September 2015 and June 2018. Informed consent was obtained from all subjects/participants for the study. The study was approved by the Stanford Hospital Institutional Review Board for research on human subjects (IRB 4344). Single-cell suspension was then achieved by dissociating the samples for 1–2 h with Collagenase/Hyaluronidase (Stemcell Technologies, 7912) in DMEM 1% FBS, followed by 2 min digestion with Trypsin–EDTA (0.25%, Life Tech, except for TuPa#1, #3 and #4). ACK and DNAse treatments were performed as needed.

Single cells were isolated by fluorescence-activated cell sorting (FACS) on FACS Aria II (BD Biosciences for TuPa#1–6) into a single well of 96-well plate and on Sony SH800Z (for TuPa 23, 27, 28, 29, 31) into single wells of 384-well Biorad HardShell plates. Antibodies used for sorting pancreatic cells were: EpCAM fluorescein isothiocyanate (FITC), CD49f phycoerythrin (PE), CD24 PE-Cy7, CD44 Allophycocyanin (APC), hCD45/GPA Pacific-blue (BioLegend). Cells were gated on the basis of forward- and side- scatter profiles, and live/dead discrimination was obtained with Sytox Blue (ThermoFisher #oS34857) or DAPI (4′,6-diamidino-2-phenylindole). The plates were pre-filled with 500 nl of lysis buffer containing poly-T capture oligos, spike-in External RNA Controls Consortium (ERCC) control RNAs, and other molecules as described elsewhere^2^. cDNA synthesis and amplification was performed as described in the same reference. Libraries using in-house Tn5 were done as described and sequenced on Illumina NovaSeq 6000 on S2 or S4 flow cells and 100 base paired-end kits at an average depth of 1 million reads per cell. To avoid index hopping, dual unique barcodes with a reciprocal Hamming distance > 2 were used.

The sequencing read pairs were mapped to the human genome using STAR RNA aligner^34^ and sorted by name. Genes were counted using HTSeq^35^. One of us (FZ) is the maintainer of HTSeq. A new package for grouping mapping and counting was developed by one of us (FZ) and is available on GitHub: https://github.com/iosonofabio/bag_of_stars. Cells with less than 100,000 reads were discarded.

### Data processing

For both datasets, count tables were further analyzed in Python 3.7 using numpy^36^, pandas^37^, and singlet. The latter package was developed by one of us (FZ) and is available on GitHub: https://github.com/iosonofabio/singlet and on PyPI: https://pypi.org/project/singlet/. A detailed description of the northstar algorithm is available in Supplementary Text 1.

## Supplementary information

Supplementary information.

## Data Availability

Northstar is available on GitHub at https://github.com/northstaratlas/northstar. Cell type averages and subsamples for a number of cell atlases are available at https://northstaratlas.github.io/atlas_landmarks/. All scripts used to generate the figures and tables for this manuscript are available on GitHub at https://github.com/northstaratlas/northstar_analysis/. The code is written in Python 3, C, and C++ and is tested via continuous integration on Linux and OSX.
