# Supplementary material for: Northstar enables automatic classification of known and novel cell types from tumor samples
Source: Sci Rep. 2020 Sep 17;10:15251. doi: 10.1038/s41598-020-71805-1 (PMC7499423; doi:10.1038/s41598-020-71805-1)
Supplement: Supplementary file 1 — Supplementary information. [file 41598_2020_71805_MOESM1_ESM.pdf]

Supplementary Materials

Northstar enables automatic classification of known and novel cell types from tumor samples

Fabio Zanini[+], Bojk A. Berghuis[+], Robert C. Jones, Benedetta Nicolis di Robilant, Rachel Yuan Nong, Jeffrey Norton, Michael F. Clarke, Stephen R. Quake

Supplementary tables and figures

Supplementary Table 1. Patient metadata for pancreatic cancer dataset

Sample	Sex	Age	Diagnosis
TuPa1	Female	58	Fibromatosis
TuPa2	Female	59	Pancreatic Ductal Adenocarcinoma (PDAC)
TuPa3	Female	59	Mucinous Cystic Neoplasm
TuPa4	Female	44	Pancreatic Ductal Adenocarcinoma (PDAC)
TuPa5	Female	68	Pancreatic NeuroEndocrine Tumor
TuPa6	Female	80	Ampullary Adenocarcinoma
TuPa 23	Female	70	Well Differentiated NeuroEndocrine Tumor
TuPa 27	Male	65	Well Differentiated NeuroEndocrine Tumor
TuPa 28	Male	52	Well Differentiated NeuroEndocrine Tumor
TuPa 29	Female	70	Invasive Adenosquamous carcinoma, g. 3
TuPa 31	Male	74	Well Differentiated NeuroEndocrine Tumor

Supplementary Table 2. Number of cells from each pancreatic tumor sample assigned to each known cell type or new cluster.

	TuPa										
**Cell type**	**1**	**2**	**3**	**4**	**5**	**6**	**23**	**27**	**28**	**29**	**31**
Macrophage							3		19		
Schwann cell											
Mast cell							1			1	
T cell							154		23	3	1
NK cell											
B cell							1				
Plasmablast							1				
Classical monocyte								1			
Nonclassical monocyte								58			
Endothelial	3										
Quiescent stellate	2	1							3	22	15
Activated stellate	105										
Ductal											
Acinar		46		16	28	2		9		3	
Alpha									1		
Beta											
Gamma/PP						1			1		
Delta									1	24	2
Epsilon									1		
19							356				
20								295			
21			18			14					201
22		1			1	1				121	
23			4		1		4		2	10	38

Supplementary Table 3. Top 15 differentially expressed genes per new cluster. Genes to which we could assign a clear association with known cell types are highlighted in boldface.

Cluster				
**19**	**20**	**21**	**22**	**23**
EGFR-AS1	APOH	PAX6	**ANXA2**	**TTR**
CTC-441N14-4	AGT	GC	C19orf33	GC
C19orf57	CFC1B	GAD2	**KRT19**	SCG5
**CD74**	SCGN	FAM159B	ANXA2P2	**PPY**
TMEM229B	TM4SF5	**KRT39**	TM4SF1	RPL34P18
**HLA-DRA**	CFC1	**PPY**	OCIAD2	**CHGB**
DNAJC27-AS1	CALY	ERO1B	TMPRSS4	PAX6
PCDH10	PCSK1N	**CHGA**	LGALS3	TM4SF4
TNR	CCL15	SCG5	PLAT	**CHGA**
GAREM2	MGST1	**TTR**	GPRC5A	ATP5EP2
RP11-253M7-1	SLC14A1	**CHGB**	S100P	C10orf10
**HLA-DPB1**	RTBDN	PAPPA2	RPL7P1	HSP90AA2P
**HLA-DRB1**	SPTSSB	CPE	RPL7P23	ERO1B
ST7L	TPPP3	SCG3	RPL7P32	PDK4
**HLA-DRB5**	**COL28A1**	PDK4	RPL7P6	TTI2

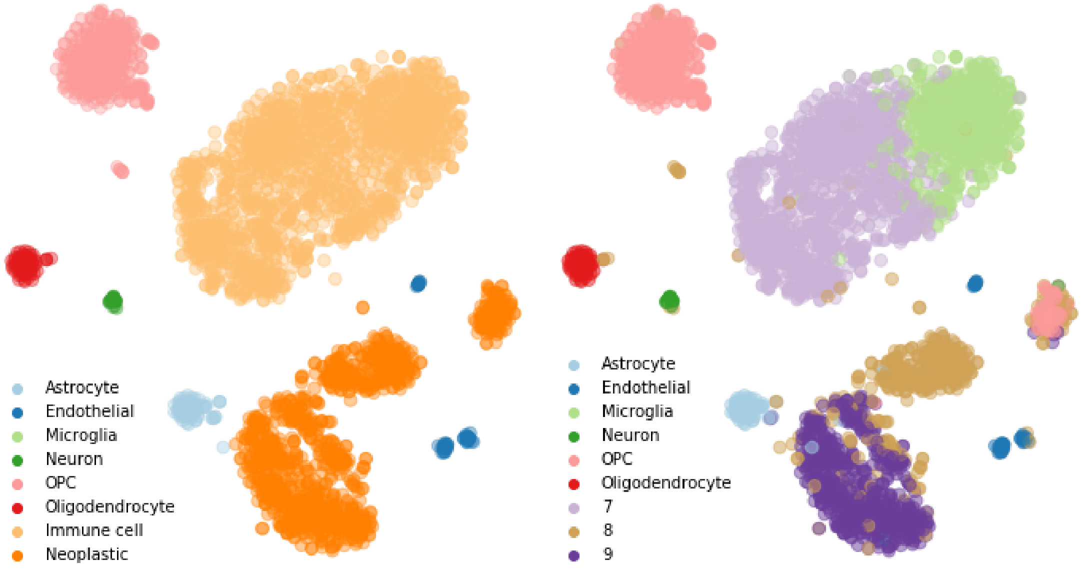

Supplementary Figure 1. Original t-SNE representation of glioblastoma dataset. Cells colored by original annotation (left) and newly annotated classes (right)

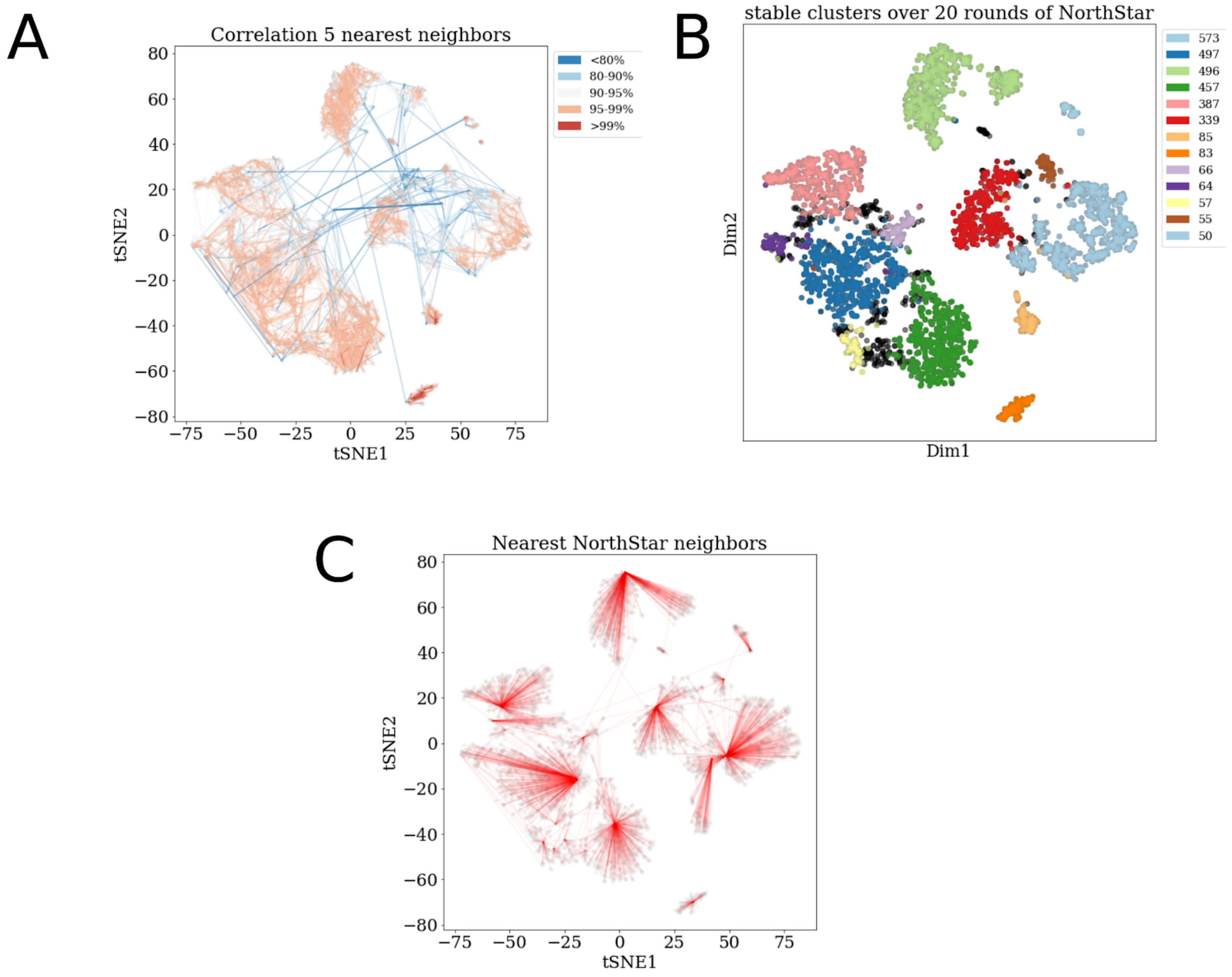

Supplementary Figure 2. Distance matrix characteristics and cluster stability using northstar. (A) Edges of 5 highest correlating cells drawn for each cell, colored by correlation (= 1 - distance). This correlation matrix of cells and atlas class 'cells' was used to perform weighted PCA and render t-SNE as shown in **Figure 2**. Correlation is the pairwise correlation of each cell using the union of top 20 overdispersed genes for each atlas class and the top 400 overdispersed genes of the new glioblastoma dataset. **(B)**: Top 11 largest clusters of cells (colors and number of cells) that always grouped together over 20 rounds of re-initialization northstar (atlas cell weight=60, number of PCs=20, resolution parameter=0.0012. threshold neighborhood=.8, with self-edging). Cells forming smaller consistent clusters or that are grouped varyingly shown in black. **(C)**: Single nearest neighbor of each cell, based on averaged class-based correlation matrix taken over 20 rounds of re-initialization of northstar.

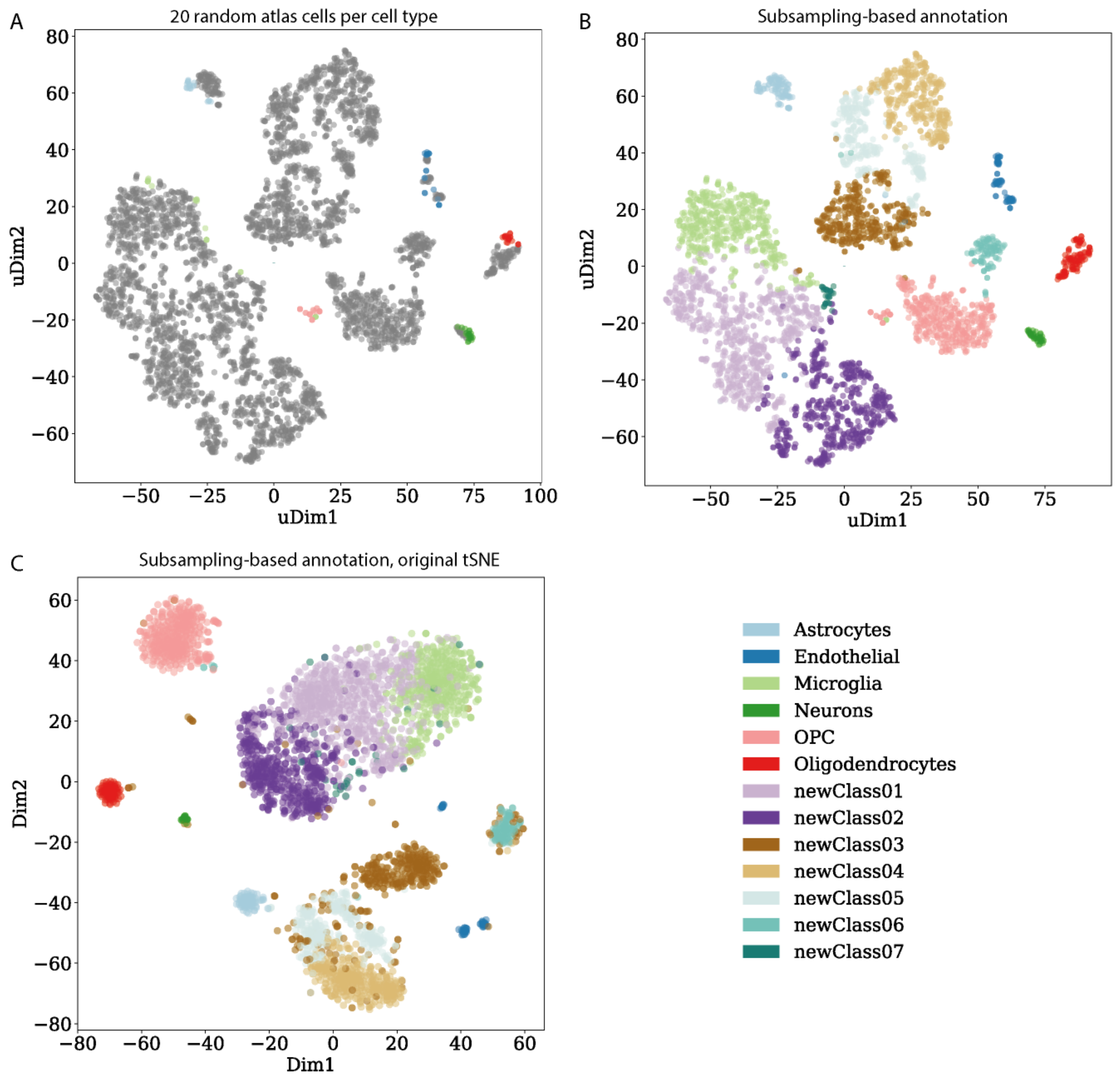

Supplementary Figure 3. Annotation of the GBM dataset by subsampling atlas cells. Notice that the microglia annotation is expanded to include around a third of the cells that were originally annotated more generically as "immune cells", in agreement with the result found by averaging within cell type.

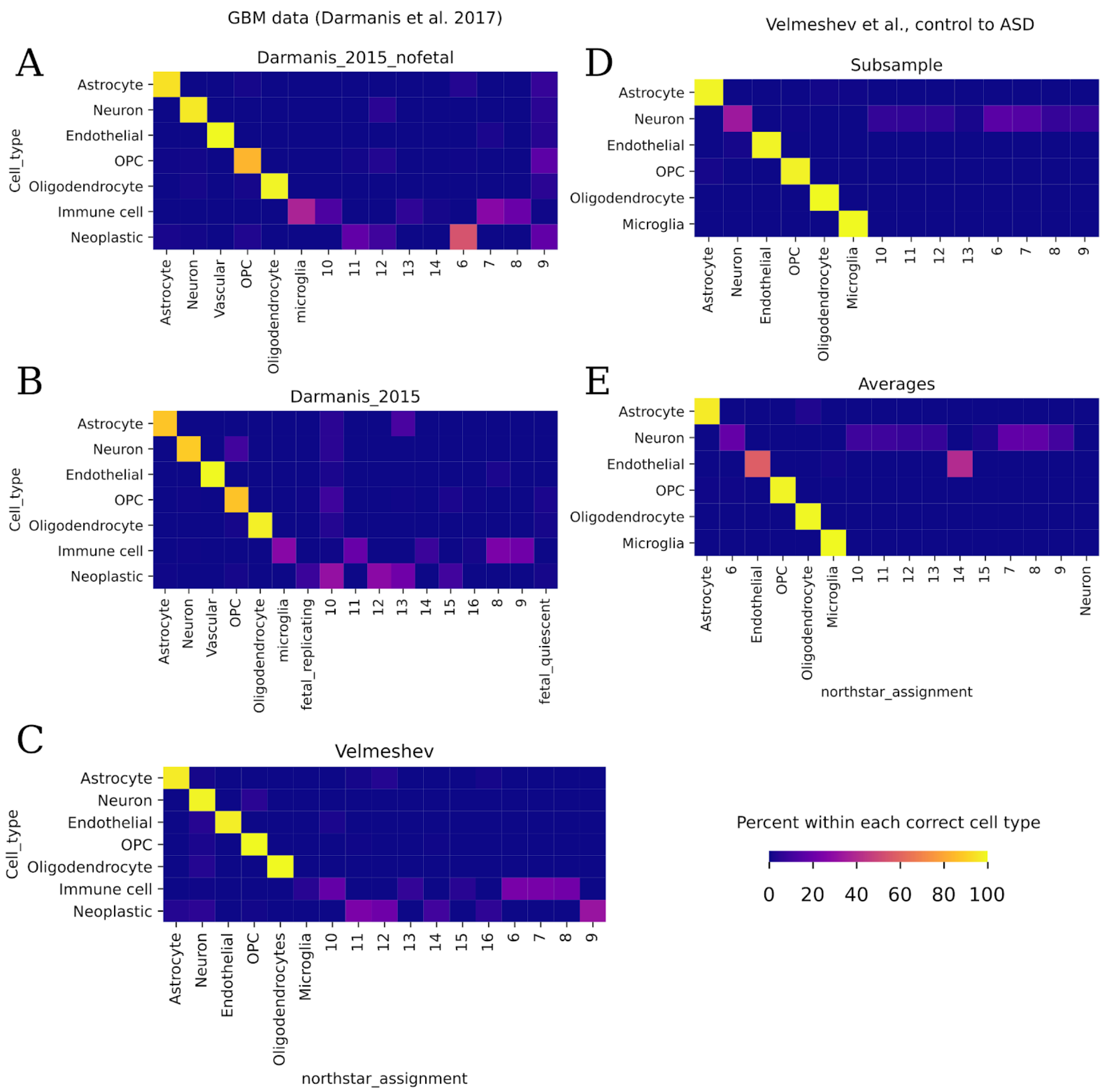

Supplementary Figure 4. Annotation of the GBM dataset and the ASD datasets. (A) Nortstar cell type assignments using the Darmanis 2015 atlas with a higher resolution parameter splits immune and neoplastic cells into subclusters. (B) Including the fetal data assigns a fraction of neoplastic cells to the cluster “fetal_quiescent”. Notice that the inclusion of developmental stages is not recommended to annotate adult tissues. (C) The usage of a distant atlas [22] based on droplet technology does not affect northstar’s accuracy. (D-E) Classifying the Velmeshev data on ASD based on their healthy samples leads to reliable classification with both northstar’s Subsample (D) and Averages (E) classes. For all panels, atlases were subsampled with 20 cells per type. For panels D and E, the ASD “new” data were sampled with 500 cells per type.

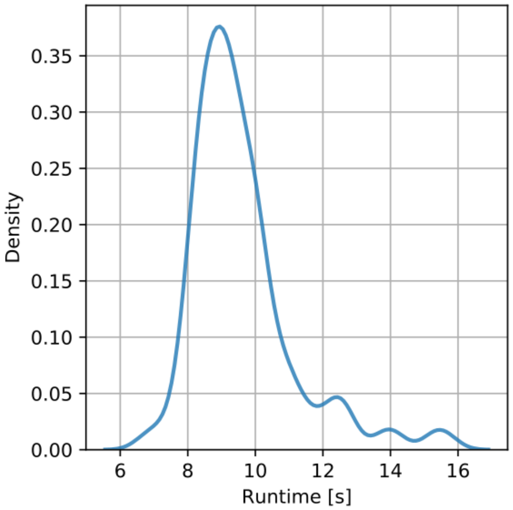

Supplementary Figure 5. Runtime performance. Distribution of northstar runtimes for the melanoma dataset (>4,000 cells) in runs with accuracy >=80% (including with default parameters).

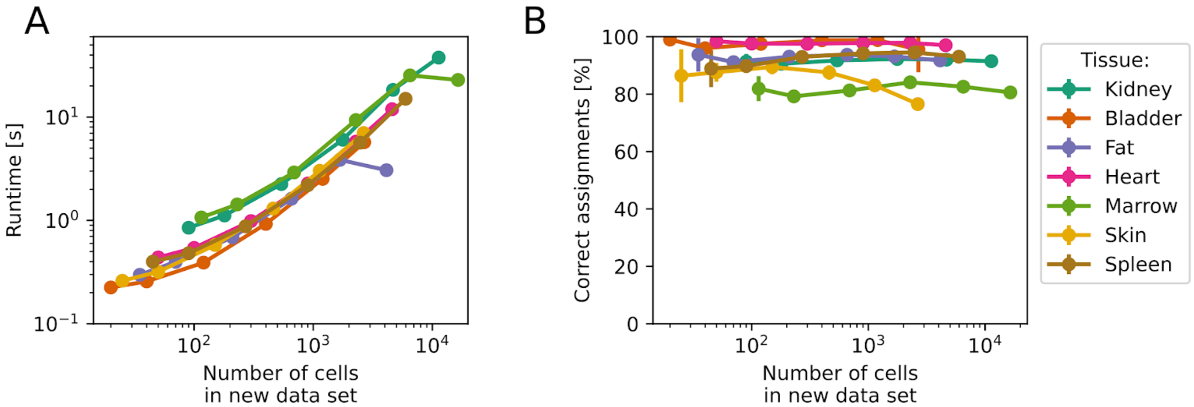

Supplementary Figure 6. (A) Runtime and (B) accuracy of northstar on Tabula Muris Senis. Droplet (10X Chromium) data from several murine tissues, using a subsample of 20 cells per type as atlas and subsamples of different sizes as test data.

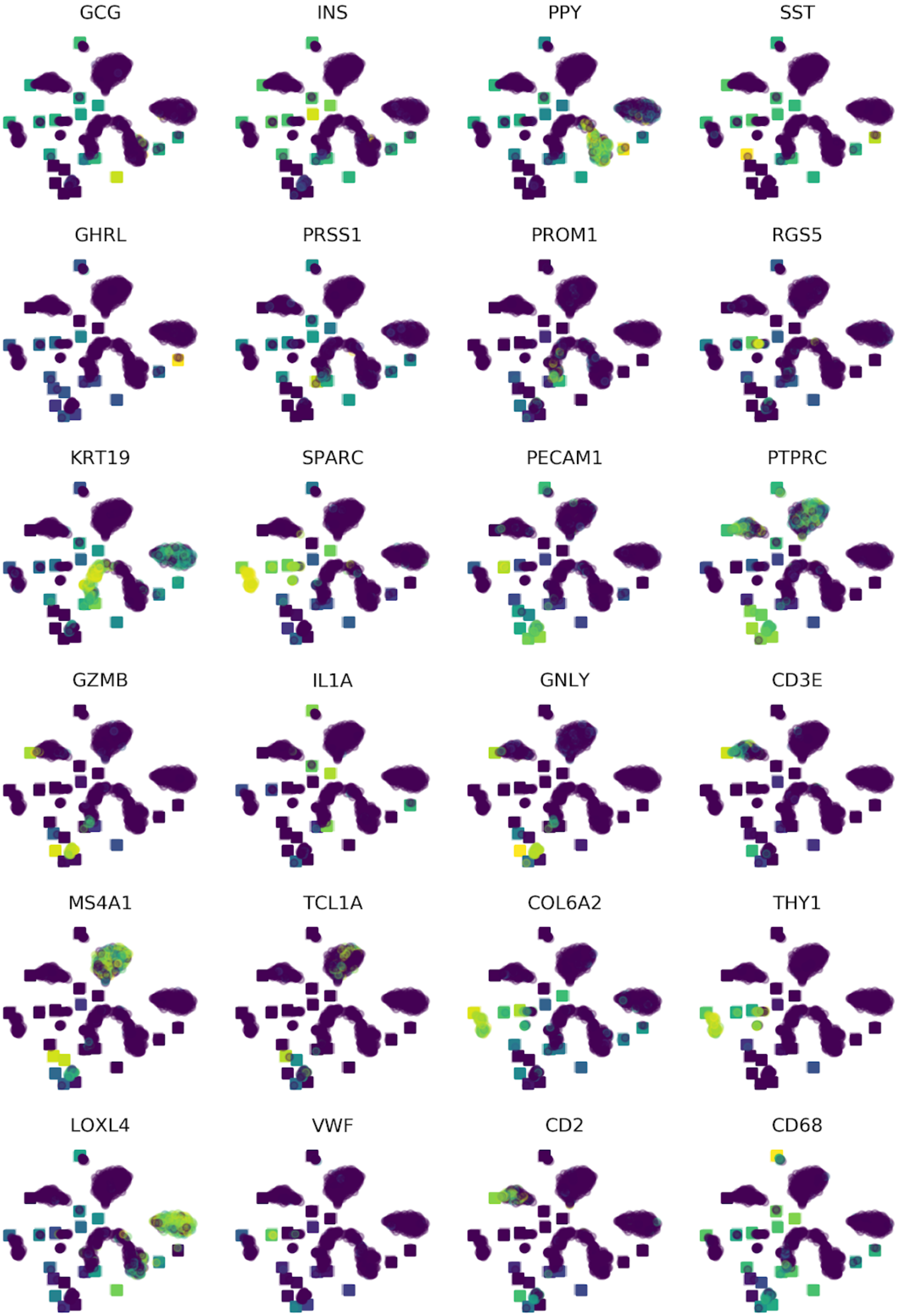
GCG
INS
PPY
SST
GHRL
PRSS1
PROM1
RGS5
KRT19
SPARC
PECAM1
PTPRC
GZMB
IL1A
GNLY
CD3E
MS4A1
TCL1A
COL6A2
THY1
LOXL4
VWF
CD2
CD68

Supplementary Figure 7. Key marker genes for different pancreatic and blood cell types projected onto the t-SNE of **Figure 3A**, colored by their expression level (low: blue, intermediate: green, high: yellow). As in **Figure 3A**, squares indicate atlas cell types, circles new cells from pancreatic tumors.

Supplementary Text 1: Detailed description of the northstar algorithm

Merge cell atlas and new single cell dataset
Northstar starts with a gene expression matrix M with N rows (cells) and L columns (genes), in agreement with the most common convention in machine learning. The first N_a rows represent the cell atlas. Since the atlas is already annotated, the gene expression of each cell can be approximated by the average of its cell type (for the Averages class) or a small subsample of cells of that type (typically 20) (for the Subsample class). Therefore, each of these rows contains the mean gene expression within a cell type from the atlas (Averages) or a representative cell of that type (Subsample).

For the Average class, in addition to these rows, the number of atlas cells within each cell type is taken as input as a vector, which we call S. S does not play a big conceptual role in the analysis, however it can skew which part of the gene space gets more weight during dimensionality reduction (see below).

The last $N_n = N - N_a$ rows of M represent single cells from the new dataset ("new cells"), which are to be annotated. For these rows, no equivalent of S is needed since each row describes only one cell. The input data for northstar is also depicted in **Figure 1A**. The steps below are illustrated in **Figure 1B**.

Only features that are shared by both atlas and the new dataset are kept. Therefore, full count matrices should be used as opposed to heavily featured selected ones.

The merged dataset is normalized by counts per million (after feature selection, see below).

Special case: merging several atlases
When more than one of the precompiled atlases are selected, the atlases need not only to be merged with the new data, but also with each other. Northstar performs this task in two steps:

1. Merging the two atlases
2. Merging the resulting composite dataset with the new cells

For step 1, several options are available. The user can take the intersection of genes between the two atlases, the union - padding the remaining atlas with zeroes - or an asymmetric join in which the features from the first atlas are all kept but the second atlas is padded with zeroes at those genes. The latter approach is the default.

The rationale behind this default is that the most common reason for an atlas missing features is that those genes were not expressed at all by the atlas cells. (A commonly used criterion is to exclude a feature from all analyses if less than 10 cells contain at least 5 or so reads/molecules.) Therefore any zero-padding is a tolerable assumption. However, in many cases one atlas will carry most of the information, so it's reasonable to ensure that the first atlas

is complete while any subsequent one can be trimmed slightly. If several atlases are key, the option ‘union’ can be used to ensure global zero padding.

Step 2 is then performed as explained above.

Feature selection

The first step of northstar is to select features (genes) to calculate the neighborhood graph. Briefly, feature selection is necessary because noisy points in a high-dimensional space are, loosely speaking, almost equidistant from one another (“curse of dimensionality”): the selection of 300-1000 most relevant genes drastically reduces noise while retaining enough information to calculate neighborhoods. It is empirically observed that excluding key features from the selection might cause overclustering, while erring on the side of too many features has a less dramatic impact on the results.

A common and simple way to select features is to take the most overdispersed genes, i.e. genes with a large Fano factor across all cells. Northstar takes this approach with the difference that overdispersion is only computed within the new dataset. In addition to those features, northstar further includes the most discriminating genes for each cell type in the atlas (largest fold change of the cell type average in a one-vs-rest comparison), to ensure that new cells can be positioned correctly within the atlas if they do belong to any of the known cell types. To maximize customizability, the user can select features before northstar if she prefers to use different criteria.

PCA (Subsample class) and weighted PCA (Averages class)

Principal component analysis (PCA) is a commonly used technique across scientific fields and is used in single cell transcriptomics to further mitigate the effect of noise in high-dimensional spaces beyond feature selection. northstar performs PCA on the feature-selected data. For the Subsample class PCA is performed normally, i.e. each atlas cell and new cell is treated in the same way.

For the Averages class, northstar uses weights to balance the influence of each atlas cell type average on the annotation result. This weighted PCA is described in the following section.

First, the weights are normalized into fraction of the total weight via division by the total number of cells in both atlas and the new dataset. The resulting vector of weights is then written in a diagonal matrix W of size N x N. We call this matrix the normalized weight matrix.

Then, M is linearly shifted to have zero weighted mean across all cells. It is then normalized by dividing by the square root of the weighted variance across all cells.

The weighted covariance matrix of M^T is calculated as $Cov_w\ M^T = M^T\ W\ M$. This matrix has dimensions L x L and is real and symmetric, so its canonical linear map has L real eigenvalues

and L real eigenvectors. Let U be the matrix with the eigenvectors as rows and the genes as columns.

The first P eigenvalues and eigenvectors are computed by standard linear algebraic techniques. Although all L components can be calculated in principle, it is in practice sufficient for the sake of subsequent steps to trim this operation to P ~ 20 because the spectrum of eigenvalues decays sharply after the first few elements.

The P eigenvectors are the top gene loadings of the weighted PCA. To find the cell loadings or principal components (PCs), we exploit the well known connection between PCA and singular value decomposition (SVD): the eigenvectors of $Cov_w\ M^T$ are also right singular vectors of the data matrix M, while the PCs are left singular vectors. The matrix V with the PCs as columns and the cells as rows can therefore be computed by matrix multiplication:

$$V = M\ U^T\ \Sigma^{-1}$$

where Σ is the smi-diagonal matrix with the singular values (i.e. the square roots of the eigenvalues of the covariance matrix) as the first diagonal entries and zero elsewhere and Σ^{-1} indicates the reciprocal of all nonzero singular values and zeros elsewhere. In theory, it is possible that this inversion cannot be computed if the covariance matrix is degenerate (i.e. it has rank < L). However, such pathological cases are unlikely to appear in biological datasets and are not considered further here.

Note that the linearity of PCA gives an intuitive explanations of why northstar works well without having to load the full atlas: having a cell type average with a weight of 200 gives almost the same dimensionality reduction as having two hundred similar cells of the same type (rows of the data matrix), while being much more efficient. Because the neighborhood graph for clustering and embedding - in northstar as in any standard scRNA-Seq approach, e.g. UMAP [33], Louvain [15], Leiden [16] - is computed in PC space which is a linear transformation away from the original data space, small heterogeneities within an atlas cell type are expected to remain small after PCA and yield a similar similarity graph to cell type averages or subsamples.

Construction of the k nearest neighbors graph

To construct the k nearest neighbors (knn) graph, a distance matrix from the new cells and all cells (including atlas and new ones) is first computed. The software package lets the user choose the distance metric and defaults onto Pearson correlation, a commonly used metric in single cell transcriptomics. The distance matrix has dimensions N_n x N.

Then, for each new cell (row in the distance matrix) the k elements at closest distance excluding self are identified. Notice that there is no need to sort the distance vector fully for this. The algorithm then scans these candidate neighbors in order of increasing distance. In the Subsample class, the k cells with shortest distance are considered neighbors. In the Averages

class, because of the weights, the situation is slightly more complex: If a candidate belongs to the new dataset, it is added to the neighbors list of this cell; If a candidate belongs to the atlas, however, it is a representative of a larger cell type which is bona fide tightly clustered around it. Hence, not only one neighbor is added but rather a number equal to the size of the cell type in the atlas. The total number of neighbors is then trimmed to k.

In addition to the neighbors from the new data into the atlas, adding a few edges that are computed outwards from the atlas helps to reduce batch effects (similarly to mutual nearest neighbors schemes). We usually compute 5 neighbors in the new dataset for each atlas average and add this small amount of edges to the similarity graph. Vice versa, northstar offers the option - which is not active by default - to force a certain number of edges *into* the atlas. This has been observed empirically to help impose a classification onto cell types that are different from but distantly related to atlas cells, e.g. cancer cell lines and their tissue of origin.

It is possible that a cell (or atlas average) has no close neighbors or just fewer than k. To consider this situation, a maximal distance threshold is used to compute the neighbor candidates. The default for correlation distance is 0.8: cells with a Pearson $r < 0.2$ with the current cell of interest are never treated as neighbors.

Once the nearest neighbors for every new cell are found, an undirected graph is constructed by symmetrization of all edges. Edges between new cells and atlas cell types can be present multiple times in the neighbors lists and are weighted accordingly. This increased edge weight ensures that if a cell is really close to a known cell type it will rapidly be absorbed into that cluster during the Leiden algorithm below. No direct edges are set between atlas cell types because they are annotated already and are not allowed to change cluster membership during the modified Leiden algorithm.

Leiden clustering with fixed nodes
The joint dataset is now represented as a neighborhood graph and can be clustered using standard graph-based methods. However, the nodes belonging to atlas cell types are already annotated and should not be allowed to change membership lest a full reannotation of the atlas is required.

Northstar solves this issue by modifying the Leiden algorithm for community detection in large graphs to allow for a number of nodes to be “fixed” into their initial membership. The Leiden algorithm itself has been proven effective in unsupervised clustering of single cell transcriptomic data, scales well with graphs with millions of nodes, and provides mathematical connectivity guarantees that lend trust to the resulting annotations.

Briefly, Leiden performs two kinds of operations on nodes, namely “move” and “merge”. Moreover, it recursively collapses the initial graph into simplified aggregated graphs in which each cluster becomes a single node: the “move” and “merge” steps are then repeated in the aggregated graph. Northstar changes both the “move/merge” steps and the aggregation step.

First, whenever a queue of nodes is constructed for consideration in terms of a move/merge, nodes that are marked as fixed are just never considered. This prevents them from switching membership to another community. Second, whenever aggregated graphs are constructed, fixed nodes are collapsed only if they belong to the same community from the beginning; otherwise they are never collapsed.

Notice that the concept of fixing the atlas nodes in the clustering step is an innovation central to northstar's design. scVI (**Figures 3B-C**) and other packages lead to much less interpretable results because they allow atlas clusters to split and mix arbitrarily, which is at odds with the annotation itself.

Output of northstar
The output of northstar is an array of each new cell's assignment to either a known cell type or a new cluster. New clusters are numbered starting from the number of known cell types upwards. For better inspection, northstar also gives access to an embedding function via t-SNE, UMAP, or PCA and to the neighborhood graph.

Supplementary Text 2: Extended methods

Analysis of the GBM dataset based on the human brain cell atlas by Darmanis et al.
First, we deleted any information on the cell type annotations on the cells from glioblastoma patients. The annotations for the cells from the healthy brain atlas, excluding fetal cells, were kept as a training set and the gene expression of the atlas was approximated by averaging within each cell type. We then performed feature selection by taking a constant number of overdispersed genes (adjustable, here 20) for each atlas cell type as well as the top overdispersed genes of the new glioblastoma cells (typically the top 500 [currently shown: top 400]) to generate a feature-selected matrix. This matrix has the dimensions of at most 5*20+400 genes by 5 cell types + 3589 new cells, though it likely has fewer genes due to genes shared between cell classes or between the atlas and the new dataset. This matrix was used to instantiate and run northstar, and for visualization purposes we created a distance matrix by calculating the pairwise correlation between cells and atlas classes, followed by a weighted PCA that gives the atlas cells the weight of 60 single cells and using the top 20 PCs to generate a 2D rendering of cell similarity using t-SNE. Projecting the original glioblastoma annotation back onto the t-SNE, we observe that our unbiased feature selection method roughly reproduces the previously reported cell clusters (**Figure 2C, Supplementary Figure 1**).

Differential expression was computed by one-vs-rest comparisons via Kolmogorov-Smirnov test on the distributions of gene expression and genes with the largest statistic (i.e. smallest P values) were identified.

Analysis of the murine melanoma dataset by Davidson et al.

Gene counts and cell metadata were downloaded from the ArrayExpress portal as specified in the original paper (experiment E-MTAB-7427). Ensembl IDs were converted into gene names as

per Ensembl Biomart, excluding ambiguous IDs. Metadata columns were renamed as needed. Cells without an assigned type were excluded. Northstar was run using the Subsample class with default parameters. The Tabula Muris bone marrow dataset has 22 immune cell types and was subsampled to 20 cells per type. Accuracy was computed on the following cell types: T cell, B cell, NK cell, monocyte, dendritic cells versus progenitor cells. Other cell types led to inconsistent unsupervised clustering and were excluded.

Analysis of the ASD brain dataset by Velmeshev et al.

To analse the Velmeshev et al. brain dataset, gene counts and cell metadata were downloaded from the UCSC portal https://cells.ucsc.edu/?ds=autism as reported in the original publication. The counts are normalized and logged already. Ensembl IDs were converted into gene names by adding all the IDs corresponding to the same gene name, as per Ensembl Biomart. The data was subsampled in several ways: 20 cells per type for most analyses were used as atlas, whereas (geometric) averages were computed among all cells. For the estimate of cell type annotation from control individuals to ASD ones, 500 cells per type were sampled from the union of all ASD invididuals. Cell types were simplified to be more consistent with the Darmanis et al (2015) atlas, in part because the finer-grained classification of Velmeshev et al, (e.g. neuronal types) led to a degree of mixture in the embeddings. Northstar was run using default parameters.
